# Taylor’s Medical Jurisprudence

**Published:** 1893-04

**Authors:** 


TAYLOR'S MEDICAL JURISPRUDENCE—Eleventh American Edition. Edited by Clark Bell Esq, Lea Bros. & Co. Philadelphia

The fact that this admirable work comes to us in its eleventh American edition, as an entire revision of all prior editions, both American and London, is of itself a sufficient testimonial of its merit. It should be in the library of every city practitioner, lawyer and chemist. In the back of the work, arranged alphabetically, are cited nearly 700 cases and authorities which should be especially valuable to attorneys in the conduct of medico-legal cases involving questions of poisoning, insanity, etc,

The work is full and complete, and gives both English and American law, so far as enacted upon experts and expert evidence. The author then takes up evidence of poisoning and the various poisons, both mineral and vegetable ; gives symptoms, effects and evidences of poisoning, with methods for analysis, and frequent plates showing microscopical appearances. This is followed by a thorough discussion of wounds and injuries ante and post-mortem appearances, blood stains and their detection ; suicide, drowning, criminal abortion, infanticide, etc. The last hundred pages of the work are devoted to the subject of insanity, giving medical and legal definitions ; laws regulating restraint and discharge of lunatics ; feigned insanity, etc.

We cannot commend the work more highly than to say that it covers the entire field of Forensic Medicine and does it thoroughly and clearly, omitting nothing that might be of service to the physician upon the witness stand, or the attorney in the preparation of his brief or examination of a witness.

